# Catalpol Ameliorates Sodium Taurocholate-Induced Acute Pancreatitis in Rats via Inhibiting Activation of Nuclear Factor Kappa B

**DOI:** 10.3390/ijms150711957

**Published:** 2014-07-04

**Authors:** Wen Qin Xiao, Guo Jian Yin, Yu Ting Fan, Lei Qiu, Xiao Feng Cang, Ge Yu, Yan Ling Hu, Miao Xing, De Qing Wu, Xing Peng Wang, Guo Yong Hu, Rong Wan

**Affiliations:** 1 Department of Gastroenterology, Shanghai Tenth People’s Hospital, Tongji University School of Medicine, 301 Yanchang Road, Zhabei District, Shanghai 200072, China; E-Mails: zhunikaixin_xwq@163.com (W.Q.X.); ygj234@163.com (G.J.Y.); sissifanyt@hotmail.com (Y.T.F.); qiuleikaoyan@163.com (L.Q.); caroline_1987@163.com (X.F.C.); lillian_hyl@126.com (Y.L.H.); xmiao0928@163.com (M.X.); sinnerwu1984@163.com (D.Q.W.); 2 Department of Gastroenterology, Shanghai First People’s Hospital, Shanghai Jiaotong University School of Medicine, 100 Haining Road, Hongkou District, Shanghai 200080, China; E-Mails: medyuge@sina.com (G.Y.); wangxingpeng@hotmail.com (X.P.W.)

**Keywords:** acute pancreatitis, catalpol, inflammatory cytokines, NF-κB

## Abstract

Catalpol, an iridoid glucoside extracted from the traditional Chinese herbal medicine, *Rehmannia glutinosa*, is reported to exert neuroprotective, anti-inflammatory, anti-tumor and anti-apoptotic effects. The main aim of the present study was to investigate whether catalpol ameliorates experimental acute pancreatitis (AP) induced by sodium taurocholate (STC). AP was induced in rats via retrograde injection of 4% STC (0.1 mL/100 g) into the biliopancreatic duct. Rats were pre-treated with saline or catalpol (50 mg/kg) 2 h before STC injection. At 12, 24 and 48 h after injection, the severity of AP was evaluated using biochemical and morphological analyses. Pretreatment with catalpol led to a significant reduction in serum amylase and lipase activities, pancreatic histological damage, myeloperoxidase (MPO) activity, interleukin (IL)-1β, IL-6 and TNF-α levels, and activation of nuclear factor kappa B (NF-κB). Moreover, administration of catalpol increased the viability of pancreatic acinar cells and inhibited NF-κB expression *in vitro*. Our results collectively support the potential of catalpol as a highly effective therapeutic agent for treatment of AP.

## 1. Introduction

Acute pancreatitis (AP) is a severe inflammatory condition of the pancreas caused by multiple factors. Although most patients with AP suffer mild disease with relatively low complication rates, ~20% of patients develop acute respiratory distress syndrome and experience multiple organ dysfunction, accompanied by a high mortality rate [1,2]. It is generally believed that the severity of pancreatitis is determined by initial events in pancreatic acinar cells. These events, including activation of zymogens and release of proinflammatory cytokines, such as interleukin (IL)-1β, IL-6 and tumor necrosis factor (TNF)-α, result in recruitment of inflammatory cells, such as neutrophils and macrophages, leading to further acinar cell injury and inflammation of chemical mediators [3,4]. Considering the vital importance of the inflammatory response in AP, effective therapy should focus on the key steps leading to this severe response. The transcription factor, NF-κB, a key regulator of cytokine induction, activates AP early on in acinar cells and promotes the expression of multiple proinflammatory genes [5,6,7,8]. NF-κB plays a vital role in progression of AP. Recent studies showed that the severity of pancreatitis in transgenic mice is increased through activation of NF-κB in acinar cells [9], and conversely, the inflammatory response and severity of AP can be attenuated through inhibition of NF-κB activity [8,10].

To date, AP treatment has been limited to the improvement of symptoms, such as relief of abdominal pain, abdominal distension, nausea and vomiting. Thus, effective new therapeutic procedures to treat AP are an urgent unmet medical need. Iridoid glycosides exist widely in plants of many families and display various biological activities, including purgative, hepatoprotective, anti-microbial, analgesic, antitumor, sedative and anti-inflammatory effects [11]. Several studies have demonstrated a broad range of biological and pharmacological effects of catalpol, an iridoid glucosideisolated from the root of *Rehmannia glutinose*, such as neuroprotection, anti-tumor [12] and anti-inflammation [13,14,15]. Research on the anti-inflammatory activity of catalpol further suggests that catalpol exerts therapeutic activity through attenuation of NF-κB activity [16,17].

Sodium taurocholate (STC), a type of bile salt, is the most widely used agent to induce AP. A rat model of STC infusion displays significantly increased serum amylase, lipase and proinflammatory cytokine levels, pancreatic edema, vacuolization, inflammation, hemorrhage, and acinar cell and fat necrosis [18]. While catalpol has been shown to affect several inflammatory diseases, its effects on AP are currently unknown. In the current study, we investigated the effects of catalpol in a rat model of STC-induced pancreatitis.

## 2. Results and Discussion

### 2.1. Results

#### 2.1.1. Preliminary Study

**Figure 1:**
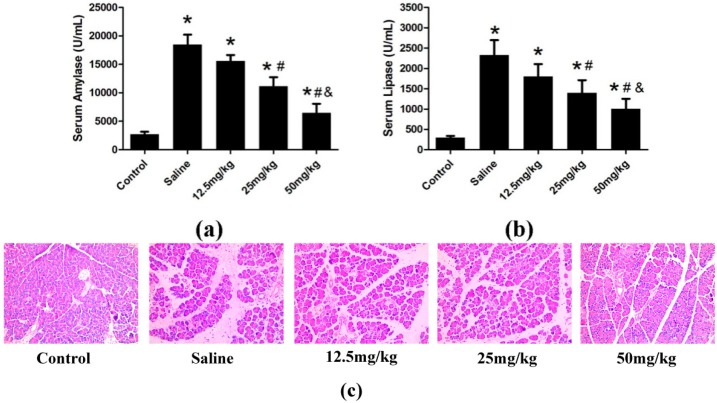
AP was induced in rats (*n* = 4 in each group) by treatment with 4% STC. Saline or catalpol (12.5, 25 and 50 mg/kg) was administered 2 h before STC injection. The control group was administered saline (0.9% NaCl) instead of STC. Rats were sacrificed 24 h after STC injection. Blood samples were collected, and levels of serum amylase (**a**) and lipase (**b**) were measured, and the staining of hematoxylin and eosin (H&E) of pancreas were showed (**c**). Original magnifications: ×200, all specimens were scored by three pathologists who were unaware of their origin. Data are presented as means ± SD from three independent experiments. * *p* < 0.05, compared with the control group; # *p* < 0.05, compared with the saline-treated group; & *p* < 0.05, compared with 25 mg/kg catalpol-treated group.

The optimal effective dose of catalpol was evaluated based on the levels of serum amylase and lipase, two biochemical indicators closely related to pancreatic damage. Moreover, we also investigated pancreas staining with hematoxylin and eosin (H&E). In our preliminary experiment, a high dose of catalpol (50 mg/kg) led to more significant decrease in serum amylase (Figure 1a) and lipase (Figure 1b), and more significant protection in pancreas histological characteristic (Figure 1c), compared with medium (25 mg/kg) and low (12.5 mg/kg) doses at 24 h after STC injection. Accordingly, 50 mg/kg was selected as the optimal dose for subsequent experiments.

#### 2.1.2. Effect of Catalpol on STC-Induced AP *in Vivo*

**Figure 2:**
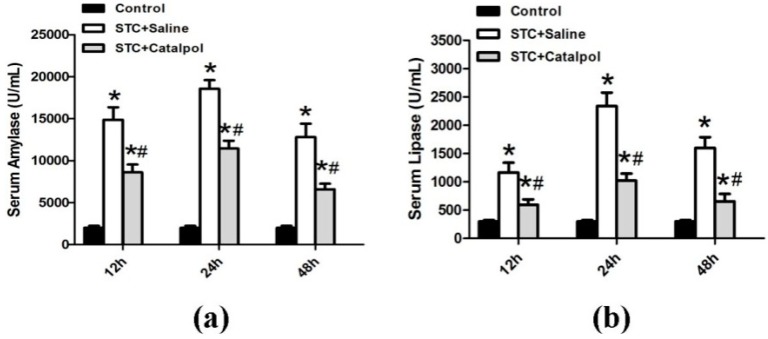
AP was induced in rats (*n* = 12 in each group) by treatment with 4% STC. Saline or catalpol (50 mg/kg) was administered 2 h before STC injection. The control group was administered saline (0.9% NaCl) instead of STC. Rats were sacrificed 12, 24 and 48 h after STC injection. Four rats were examined at every time-point in each group. Blood samples were collected, and activities of serum amylase (**a**) and lipase (**b**) were measured. Data are presented as means ± SD of three independent experiments. * *p* < 0.05, compared with the control group at the same time-point; # *p* < 0.05, compared with the STC + saline-treated group at the same time-point.

After retrograde injection of 4% STC into the biliopancreatic duct, we evaluated the severity of AP by measuring serum amylase and lipase levels, the most commonly used biochemical indicators of AP. Notably, catalpol induced a significant reduction in the levels of amylase (Figure 2a) and lipase (Figure 2b) in rat serum.

**Figure 3:**
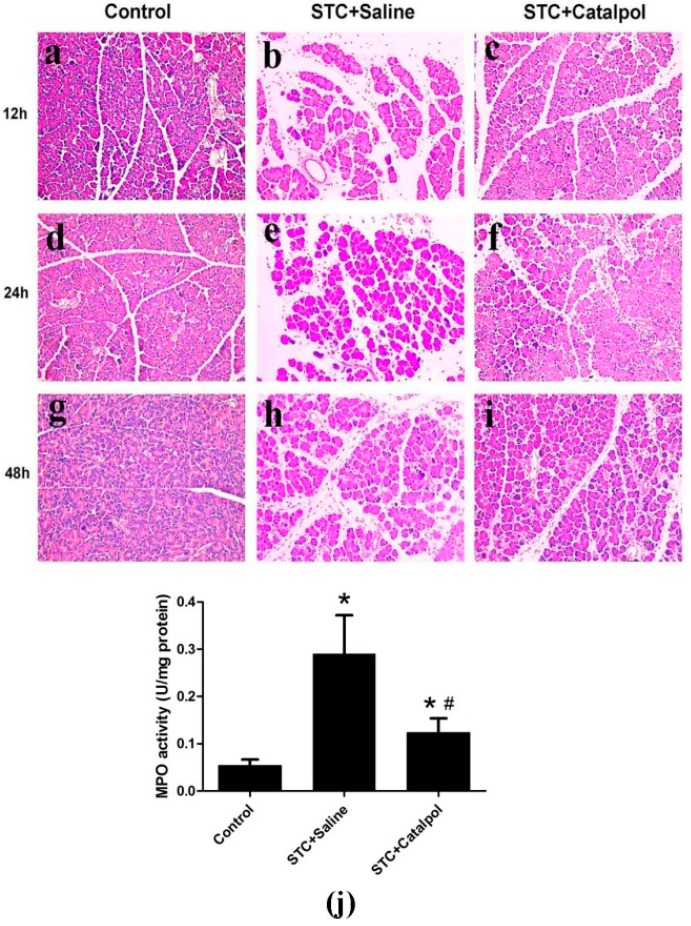
Representative hematoxylin and eosin (H&E)-stained sections of pancreas are shown. Control group at each time-point: (**a**,**d**,**g**); STC + saline-treated group at each time-point: (**b**,**e**,**h**); STC + catapol-treated group at each time-point: (**c**,**f**,**i)**; Original magnification: ×200, all specimens were scored by three pathologists who were unaware of their origin. MPO activity was measured 24 h after STC injection (**j**). Data are presented as means ± SD of three independent experiments. * *p* < 0.05, compared with the control group; # *p* < 0.05, compared with the STC + saline-treated group.

To establish the effects of catalpol on development and severity of AP, rats were pretreated with saline or catalpol (50 mg/kg) before STC injection. In normal rats, the histological features of the pancreas were typical of normal architecture (Figure 3a,d,g). Histological examination of the pancreas at 12, 24 and 48 h after STC injection revealed tissue damage characterized by inflammatory cell infiltration and acinar cell necrosis (Figure 3b,e,h). Pretreatment with catalpol resulted in significant reduction of pancreatic injury, edema, inflammation, vacuolization and necrosis, compared with the STC + saline-treated group (Figure 3c,f,i). Catalpol additionally suppressed the activity of MPO at 24 h after STC injection (Figure 3j).

**Figure 4:**
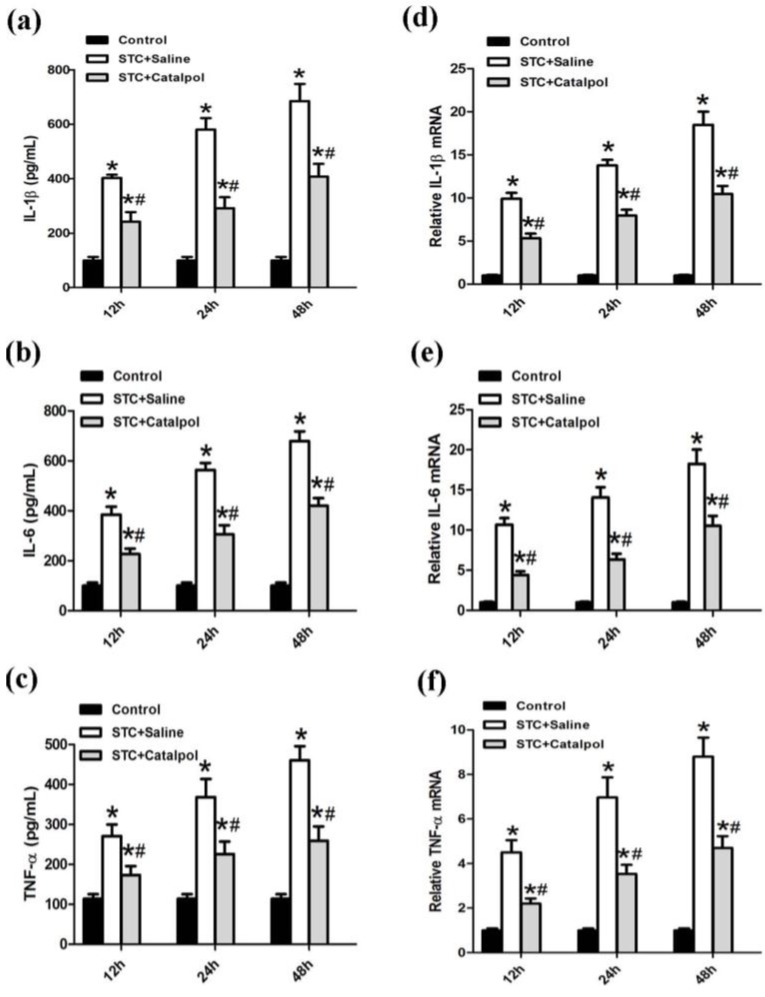
Serum proinflammatory cytokines, such as IL-1β, IL-6 and TNF-α were measured using ELISA (**a**,**b**,**c**); IL-1β, IL-6 and TNF-α mRNA levels were detected using quantitative RT-PCR (**d**,**e**,**f**) using GAPDH mRNA as the housekeeping control. Data are presented as means ± SD from three independent experiments. * *p* < 0.05, compared with the control group at the same time-point; # *p* < 0.05, compared with the STC + saline-treated group at the same time-point.

Activation of NF-κB in rat AP enhanced the release of several proinflammatory cytokines, including IL-1β, IL-6 and TNF-α. Upon pretreatment with catalpol, production of proinflammatory cytokines in serum, and mRNA expression levels were significantly reduced (Figure 4a–f).

**Figure 5:**
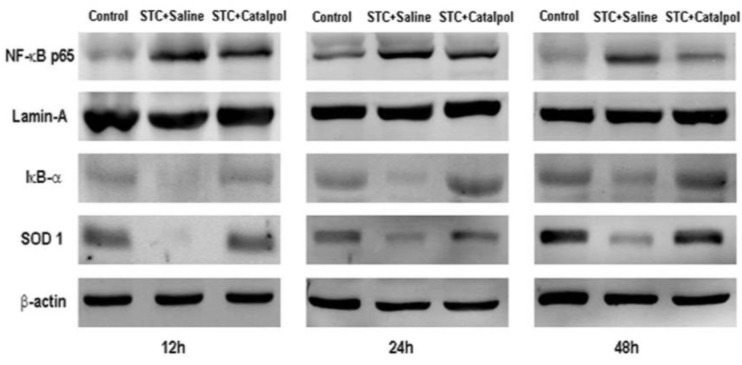
Western blot analysis of NF-κB p65 in the nucleus, and IκB-α and SOD1 in the cytoplasm or whole protein at three time-points for each group. Lamin A and β-actin were used as the internal references for nuclear and cytoplasmic or whole proteins, respectively. Figures are representative of three independent experiments.

**Figure 6:**
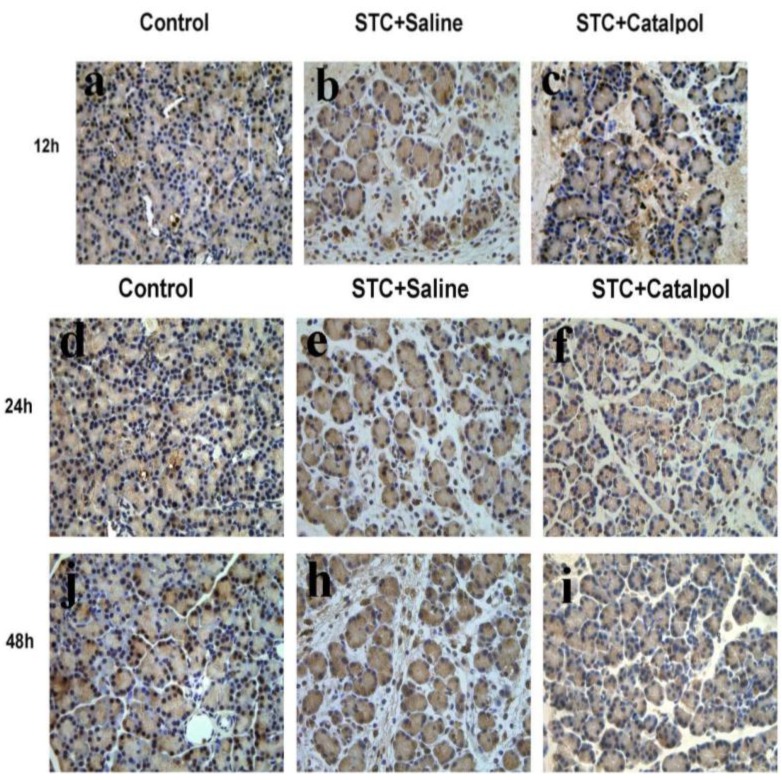
Immunohistochemical analysis to detect NF-κB expression at three time-points in each group. Control group at three time-points (**a**,**d**,**g**); STC+saline-treated group at the three time-points (**b**,**e**,**h**); STC+catalpol-treated group at the three time-points (**c**,**f**,**i**). Original magnification: ×400, all specimens were observed by three pathologists unaware of their origin. Results are representative of three independent experiments.

Activation of NF-κB plays an important role in the induction of proinflammatory mediators. Nuclear translocation of the NF-κB transcription factor is preceded by the degradation of inhibitory κB (IκB)-α and superoxide dismutase 1 (SOD1). To determine whether catalpol effects NF-κB activity, we examined NF-κB p65 nuclear translocation, as well as IκB-α and SOD1 expression via western blot and immunohistochemistry. Catalpol enhanced IκB-α and SOD1 levels, blocked nuclear NF-κB p65 expression during STC-induced AP (Figure 5), and induced degradation of NF-κB p65 (as evident from the staining intensity) in the nucleus, particularly at 12 h after STC injection (Figure 6a–j).

#### 2.1.3. Effect of Catalpol on STC-Induced AP *in Vitro*

**Figure 7:**
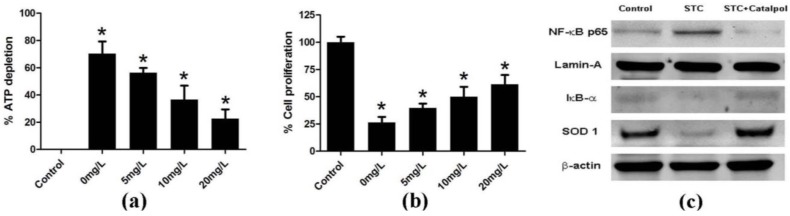
Acinar cell necrosis was determined by measuring ATP levels using luciferin/luciferase-based methods. Catalpol suppressed the dose-dependent depletion of ATP in acinar cells induced by infusion of STC (**a**); and increased dose-dependent proliferation of acinar cells by CCK-8 (**b**); Expression of NF-κB p65 in the nucleus, and IκB-α and SOD 1 in the cytoplasm of pancreatic acinar cells was detected via western blot. Lamin-A and β-actin were used as the internal references for nuclear and cytoplasmic proteins, respectively (**c**). Data are presented as means ± SD of three independent experiments. * *p* < 0.05, compared with the control group.

ATP depletion is associated with pancreatic acinar cell necrosis [19]. Cell Counting Kit-8 is a sensitive colorimetric assay that can be applied to detect acinar cell proliferation. Here, the viability of isolated acinar cells incubated with or without STC (3750 nmol/L) and catalpol at different doses (0, 5, 10, 20 mg/L) was analyzed with the Cell Titer-Glo Luminescent Cell Viability Assay kit and Cell Counting Kit-8 (CCK-8). As expected, catalpol improved the viability of pancreatic acinar cells (Figure 7a,b), and suppressed NF-κB activity (Figure 7c).

### 2.2. Discussion

AP is a common inflammation disorder with increasing incidence trends over recent years [20]. AP is characterized by edema, inflammation, vacuolization and necrosis, and commonly caused by biliary tract disease, excessive ethanol consumption, certain medications, and invasive procedures of the biliary and pancreatic ducts [21,22,23,24]. The mechanism underlying the progression of AP is poorly understood and the clinical course is unpredictable. Therefore, identification of effective therapeutic options to treat AP is an urgent medical need. Inflammation mediators, such as the proinflammatory cytokines, IL-1β, IL-6 and TNF-α are involved in the development of pancreatitis and play a pivotal role in systemic manifestations besides modulating pancreatic acinar cell injury, reducing the cascade of cytokines in the early stages and ameliorating the disease and its systemic complications [3,25]. In earlier experimental AP studies, serum levels of IL-1β and TNF-α was elevated, and conversely, their blockade attenuated the progression of disease [25,26]. IL-6 is a principal cytokine mediator of the acute-phase response, and proposed as a marker for predicting the severity of AP [25]. The level of IL-6 in severe pancreatitis is markedly higher than that in mild pancreatitis [27]. Suppression of expression of these proinflammatory cytokines is reported to ameliorate the severity of pancreatitis [28]. MPO, a type of peroxidase secreted by neutrophils, is used to detect neutrophil activity [29]. In the present study, we examined the effect of catalpol in a well-characterized model of AP induced by STC, which is similar to human AP in view of the typical inflammation performance. Our results collectively demonstrate that catalpol significantly ameliorates damage from pancreatitis, as evident from the reduction of serum amylase and lipase activities, improvement in histological features, decrease in MPO activity, and lower IL-1β, IL-6 and TNF-α expression in serum and mRNA. 

Accumulating evidence has shown effects of catalpol on related inflammatory disorders, such as neurodegenerative diseases associated with inflammation [13,14,17,30]. Catalpol exerts its therapeutic effects through several mechanisms, including attenuation of nitric oxide increased via ERK signaling pathways induced by rotenone in mesencephalic neurons [31], blocking hydrogen peroxide-induced apoptosis by activating the PI3K/Akt-Bad signaling pathway, increasing Bcl-2 and decreasing Bax expression [32,33]. Catalpol has been shown to have an inhibitory effect on the inflammatory reaction in astrocytes, with inactivation of NF-κB proposed as the major determinant for its anti-inflammatory mechanism [17]. Moreover, catalpol suppresses AGE-mediated inflammation by inhibiting ROS production and NF-κB activity [34].

NF-κB is a pleiotropic transcription factor with key functions in the intestinal immune system. The family members control the transcriptional activities of various promoters of proinﬂammatory cytokines, cell surface receptors, transcription factors, and adhesion molecules involved in intestinal inﬂammation. NF-κB is located in the cytoplasm of most cells as an inactive complex with unprocessed precursor proteins (such as p105) or IκB (such as IκB-α proteins). Degradation of these precursor proteins enables NF-κB dimers to enter the nucleus and activate specific target gene expression. The transcription factor plays an essential role in the transcriptional regulation of cytokines, such as IL-1β, IL-6 and TNF-α [35]. Activation of the NF-κB pathway promotes inflammatory parameters associated with severe AP, strongly suggesting that NF-κB in pancreatic acinar cells is a major proinflammatory factor in the development of pancreatitis and systemic inflammatory response [36]. Inhibition of NF-κB has been shown to improve survival in rats with taurocholate-induced pancreatitis [7]. In view of these findings, development of NF-κB inhibition strategies to treat AP has generated significant attention. Here, we investigated whether catalpol ameliorates the severity of AP via suppression of NF-κB activity. Moreover, since activation of NF-κB is an early event in AP, NF-κB activity was investigated at different time-points of 12, 24 and 48 h. Our findings demonstrated that catalpol significantly suppresses degradation of IκB-α, leading to attenuation of NF-κB p65 expression in the nucleus during STC-induced AP. Immunohistochemistry results showed that catalpol decreases the staining intensity of nuclear NF-κB p65, which was particularly evident at the 48 h time-point after STC injection. In addition, catalpol improved the severity of pancreatitis *in vitro* through increasing the viability of acinar cells and inhibiting NF-κB activation. SOD is an important free radical scavenger, which specifically detoxifies superoxide radicals to hydrogen peroxide and protects against free radical attack. SOD1, a crucial member of the SOD family, reduces neuron loss and diminishes the area of injury in the acute phase of post-ischemia [37]. In this study, western blot experiments revealed that catalpol can increase the expression of SOD1, thus demonstrated that catalpol may have antioxidant effect. However, the complete mechanism of catalpol antioxidant effect requires further investigation.

## 3. Experimental

### 3.1. Animals and Main Materials

Male Sprague-Dawley rats were purchased from Shanghai SLAC Laboratory Animal Co., Ltd. (Shanghai, China). Animals were maintained under 12 h light–dark cycles at 22 °C, provided water *ad libitum*, fed standard laboratory chow, and allowed to acclimatize for a minimum of one week. The environment was maintained at a relative humidity of 30%–70%. Rats weighing 250 ± 30 g were randomly assigned to control or experimental groups. All animal-related procedures were approved by the Animal Care and Use Committee of The Tenth People’s Hospital of Shanghai, Tongji University. This research was approved by the Science and Technology Commission of Shanghai Municipality (ID: SYXK 2007-0006) under the permit number 2011-RES1. Purified catalpol (98%) was purchased from Tauto Biotech (Shanghai, China). Sodium taurocholate (STC), eosin and hematoxylin, lamin-A and β-actin were acquired from Sigma Chemical (Sigma-Aldrich, St. Louis, MO, USA), and antibodies against NF-κB p65, inhibitory κB (IκB)-α and superoxide dismutase 1 (SOD 1) from Abcam (Hong Kong, China). Unless stated otherwise, all other chemicals were purchased from Sigma.

### 3.2. Induction of Experimental Design

Prior to the experiment, catalpol was dissolved in saline (0.9% NaCl) and filtered with a 0.22 μm colander. To obtain the optimal dose of catalpol for preventing AP, induced via retrograde injection of 4% STC into the biliopancreatic duct [38], a preliminary study was performed. Sixteen rats were randomly divided into four groups: Group 1, saline-treated; Groups 2, 3 and 4, catalpol-treated (12.5, 25 and 50 mg/kg, *per os* (p.o)), respectively. Pretreatment with saline or catalpol was performed 2 h before STC injection. Rats were sacrificed 24 h after saline or STC injection, at which time pancreatic damage had already peaked. Blood samples were collected to detect the levels of serum amylase and lipase, two biochemical indicators closely related to pancreatic damage. The optimal dose of catalpol was consequently established as 50 mg/kg and used for the next series of experiments. Thirty-six rats were randomly divided into three groups (Group 1, normal control; Group 2, STC + saline-treated; Group 3, STC + Catalpol-treated) and fasted overnight with continued access to water. Induction of experimental AP and administration of saline or catalpol was performed in a similar manner as described for the preliminary experiment. Rats were sacrificed under anesthesia at all three time-points (12, 24 and 48 h) after induction of AP. The pancreas was rapidly removed from each rat, a portion fixed in 4% paraformaldehyde buffered with phosphate-buffered saline (PBS) overnight at 4 °C, and embedded in paraffin wax or frozen immediately at −80 °C. The remaining portion was quickly ground into liquid nitrogen and frozen at −80 °C until further use. Blood samples were maintained at room temperature for 2 h before centrifugation (~3000*× g*) at 4 °C for 15 min, and serum stored at −80 °C. Amylase, lipase and cytokine levels in serum were evaluated.

### 3.3. Isolation of Pancreatic Acinar Cells

Acinar cells were isolated from rat pancreas using a previously described collagenase digestion procedure [39]. For treatment, freshly isolated acinar cells were cultivated at 37 °C, 5% CO_2_ in Dulbecco’s modified Eagle’s medium/Ham F-12 (Gibco BRL, Middleton, WI, USA) containing 10% fetal bovine serum (FBS; Gibco) and 1% penicillin–streptomycin (Gibco) with or without STC, catalpol at different doses (0, 5, 10 and 20 mg/L), and other agents, as described for the relevant experiments.

### 3.4. Determination of Serum Amylase, Lipase and Serum Proinflammatory Cytokines

Serum activities of amylase and lipase were measured via enzyme dynamics chemistry using commercial kits in a Roche/Hitachi modular analytics system (Roche, Mannheim, Germany), according to the manufacturer’s protocols. A commercial enzyme-linked immunosorbent assay (ELISA) kit (Quantikine, R&D Systems, Minneapolis, MN, USA) was used for measuring the levels of serum IL-1β, IL-6 and TNF-α.

### 3.5. Histological Examination

A proportion of pancreatic tissue was fixed in 4% phosphate-buffered formaldehyde in 24 h, dehydrated via a graduated ethanol series, and embedded in paraffin blocks. Pancreatic sections (5 μm) were dewaxed in xylene, hydrated through an upgraded ethanol series, and stained with hematoxylin and eosin. Morphological changes were examined under a light microscope by three pathologists who were unaware of the original specimens.

### 3.6. Measurement of Myeloperoxidase Activity

Neutrophil sequestration in the pancreas was quantified by measuring tissue myeloperoxidase (MPO) activity [40,41]. Pancreatic tissue samples were thawed, homogenized in phosphate buffer (20 mM, pH 7.4), and centrifuged at 4 °C for 10 min at ~10,000*× g*. The resulting pellet was resuspended in phosphate buffer (50 mM, pH 6) containing 0.5% hexade cyltrimethylammonium bromide. The suspension was subjected to four cycles of freeze-thaw, and further disrupted via sonication for 40 s. Following centrifugation of the sample at 4 °C for 5 min at ~10,000*× g*, the supernatant was used for the MPO assay. The reaction mixture contained the supernatant, tetramethylbenzidine (1.6 mM) and hydrogen peroxide (0.3 mM), which were prepared in sodium phosphate buffer (80 mM, pH 5.4). After incubation at 37 °C for 110 s, the reaction was terminated with H_2_SO_4_ (2 M), and absorbance measured at 450 nm for 5 min using a Beckman spectrophotometer (Beckman DU 640B, San Diego, CA, USA). One unit of MPO activity was defined as that degrading peroxide (1 mM) at 25 °C per min. Activity was expressed in units per milligram of tissue.

### 3.7. Quantitative Real-Time PCR

Total RNA was extracted from pancreatic tissue using TRIzol reagent (Invitrogen, CA, USA) following the manufacturer’s instructions, and subjected to reverse transcription using the PrimeScript RT reagent Kit (TaKaRa, Otsu, Japan). Quantitative real-time PCR (qRT-PCR) was performed in triplicate for each gene of interest using the ABI Prism 7900 HT Sequence Detection System (Applied Biosystems, Carlsbad, CA, USA) according to the SYBR Premix EX Taqmanual (TaKaRa). GAPDH was used as a separate endogenous control to which the gene of interest was normalized, and fold change for gene expression levels calculated using the comparative *C*_t_ (2^−∆∆*C*t^) method. Primer sequences for the biomarkers were designed with software as follows: rat IL-1β (forward, CTTCAAATCTCACAGCAGCATC; reverse, GCTGTCTAATGGGAACATCACA), rat IL-6 (forward, TCCGTTTCTACCTGGAGTTTGT; reverse, GTTGGATGGTCTTGGTCCTTAG); rat TNF-α (forward CATGGATCTCAAAGACAACCAA; reverse, CTCCTGGTATGAAATGGCAAAT), and rat GAPDH (forward, CGTATCGGACGCCTGGTTA; reverse, ACTGTGCCGTTGAACTTGC).

### 3.8. Western Blot Analysis

For western blot, rat pancreatic tissues were retrieved from storage and rapidly ground in liquid nitrogen. The resulting powder or isolated acinar cells were lysed in using a nuclear and cytoplasmic protein extract kit (Beyotime, Beijing, China), following the manufacturer’s protocol, for preparation of nuclear and cytoplasmic proteins (Pierce, CA, USA). Whole proteins of pancreatic tissues or isolated acinar cells were reconstituted in ice-cold RIPA buffer containing phenylmethanesulfonyl fluoride (PMSF, 1 mM) and a cocktail of protease inhibitors (1:100 dilution; Sigma-Aldrich), and homogenates of pancreatic tissues centrifuged at 4 °C for 15 min at ~12,000*× g*. Acinar cell supernatants were centrifuged in a ultrafiltration tube (Vivaspin 20, 3000 MWCO PES, Sartorius, Germany) at 4 °C for 100 min at ~4000*× g*. Concentrations of nuclear, cytoplasmic and whole proteins were determined using the BCA method (Pierce, Rockford, LA, USA). A 80 µg aliquot of protein or equal proportion of concentrated supernatant was subjected to sodium dodecyl sulfate/polyacrylamide gel electrophoresis (SDS-PAGE Bio-Rad, Hercules, CA, USA) and transferred to nitrocellulose/PVDF membrane following the standard method. Non-specific binding to the membrane was blocked with 5% (*w*/*v*) dry non-fat milk in Tris-buffered saline/0.05% Tween-20 (TBST) at room temperature for 1 h in a covered container. Blots were incubated overnight at 4 °C with rabbit polyclonal anti-NF-κB p65 antibody (1:1000), rabbit polyclonal anti IκB-α antibody (1:200), rabbit polyclonal anti-SOD1 antibody (1:500 dilution), rabbit polyclonal anti-Lamin-A (1:500 dilution), and mouse monoclonal anti-β-actin (1:1000) diluted in 5% bovine serum albumin (BSA). Lamin-A and β-actin were used as the internal references for nuclear and cytoplasmic, whole proteins, respectively. Membranes were washed with TBST and incubated with a secondary goat anti-rabbit IgG-horseradish peroxidase (HRP) antibody (1:2000) or goat anti-mouse IgG-HRP antibody (1:2000) obtained from Santa Cruz Biotechnology (Santa Cruz, CA, USA) diluted in 5% (*w*/*v*) dry non-fat milk in TBST for 1 h at room temperature. Finally, membranes were washed with TBST, developed using the ECL detection system (Santa Cruz Biotechnology), dried, and exposed to ECL film.

### 3.9. Immunohistochemistry

Formalin-fixed, paraffin-embedded samples were cut to 5 μm thickness. Each tissue section was deparaffinized and rehydrated with upgraded ethanol. For antigen retrieval, slides were boiled in EDTA (1 mM, pH 8.0) for 15 min in a microwave oven. Endogenous peroxidase activity was quenched with 0.3% hydrogen peroxide solution for 10 min at room temperature. After rinsing with PBS, slides were blocked with BSA in PBS for 30 min. Slides were subsequently incubated with a polyclonal antibody against NF-κB p65 (1:100) overnight at 4 °C. Antibody binding was detected with an Envision Detection Kit, Peroxidase/DAB, Rabbit/Mouse (Gene Tech, Shanghai, China). Sections were counterstained with hematoxylin. For NF-κB p65 in control status, (Please confirm) the cytoplasm of positive cells was stained, and translocation of positive cells to nuclei from the cytoplasm indicated activation of NF-κB p65. Positive areas stained with NF-κB p65 were observed in all specimens under a microscope at a magnification of ×400 by three pathologists who were unaware of specimen origins (CTR 6000; Leica, Wetzlar, Germany).

### 3.10. Quantification of Cell Viability

Cell viability was determined by measuring ATP levels using the Cell Titer-Glo Luminescent Cell Viability Assay kit (Promega, Madison, WI, USA) [42] and proliferation of acinar cells detected using the Cell Counting Kit-8 (Dojindo, Kumamoto, Japan), according to manufacturer’s instructions. 

### 3.11. Statistical Analysis

Results were expressed as means ± standard deviation (SD). Statistical analysis was performed using one-way ANOVA, followed by Student–Newman–Keuls (SNK) as a *post hoc* test. The Kruskal-Wallis test was used to evaluate the differences in categorical values, followed by Mann-Whitney U tests as a *post hoc* test. Data were accepted as statistically significant at *p* < 0.05.

## 4. Conclusions

In summary, catalpol ameliorates the severity of AP through inhibiting the release of proinflammatory cytokines and activation of NF-κB *in vivo*. Our results further indicate that administration of catalpol increases the viability of pancreatic acinar cells and inhibits NF-κB expression *in vitro*. Inhibition of NF-κB activation is part of the mechanism underlying the protective effects of catalpol against AP. The newly identified therapeutic role of catalpol in AP should provide a basis for further experimental investigation and clinical studies of this condition.

## Figures and Tables

**Figure A1:**
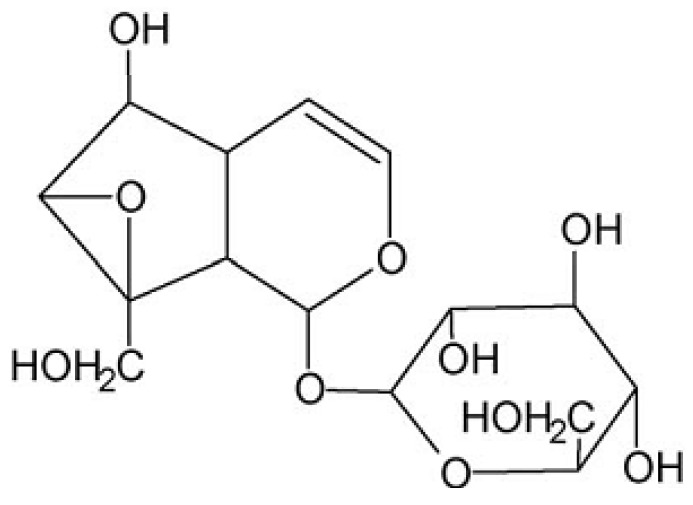
The chemical structure of catalpol. Catalpol, an iridoid glucoside extracted from the traditional Chinese herbal medicine, *Rehmannia glutinosa*, with a small molecular weight of 362.4.
